# Dysregulated CXCR4 expression promotes lymphoma cell survival and independently predicts disease progression in germinal center B-cell-like diffuse large B-cell lymphoma

**DOI:** 10.18632/oncotarget.3343

**Published:** 2015-01-21

**Authors:** Jiayu Chen, Zijun Y. Xu-Monette, Lijuan Deng, Qi Shen, Ganiraju C. Manyam, Azahara Martinez-Lopez, Li Zhang, Santiago Montes-Moreno, Carlo Visco, Alexandar Tzankov, Lihui Yin, Karen Dybkaer, April Chiu, Attilio Orazi, Youli Zu, Govind Bhagat, Kristy L. Richards, Eric D. Hsi, William W.L. Choi, J. Han van Krieken, Jooryung Huh, Maurilio Ponzoni, Andrés J.M. Ferreri, Xiaoying Zhao, Michael B. Møller, John P. Farnen, Jane N. Winter, Miguel A. Piris, Lan Pham, Ken H. Young

**Affiliations:** ^1^ Medical School of Taizhou University, Taizhou, Zhejiang, China; ^2^ Department of Hematopathology, The University of Texas MD Anderson Cancer Center, Houston, TX, USA; ^3^ Department of Bioinformatics and Computational Biology, The University of Texas MD Anderson Cancer Center, Houston, TX, USA; ^4^ Hospital Universitario Marques de Valdecilla, Santander, Spain; ^5^ San Bortolo Hospital, Vicenza, Italy; ^6^ University Hospital, Basel, Switzerland; ^7^ Aalborg University Hospital, Aalborg, Denmark; ^8^ Memorial Sloan-Kettering Cancer Center, New York, NY, USA; ^9^ Weill Medical College of Cornell University, New York, NY, USA; ^10^ The Methodist Hospital, Houston, TX, USA; ^11^ Columbia University Medical Center and New York Presbyterian Hospital, New York, NY, USA; ^12^ University of North Carolina School of Medicine, Chapel Hill, NC, USA; ^13^ Cleveland Clinic, Cleveland, OH, USA; ^14^ University of Hong Kong Li Ka Shing Faculty of Medicine, Hong Kong, China; ^15^ Radboud University Nijmegen Medical Centre, Nijmegen, Netherlands; ^16^ Asan Medical Center, Ulsan University College of Medicine, Seoul, Korea; ^17^ San Raffaele H. Scientific Institute, Milan, Italy; ^18^ Zhejiang University School of Medicine, Second University Hospital, Hangzhou, China; ^19^ Odense University Hospital, Odense, Denmark; ^20^ Gundersen Lutheran Health System, La Crosse, WI, USA; ^21^ Feinberg School of Medicine, Northwestern University, Chicago, IL, USA; ^22^ The University of Texas School of Medicine, Graduate School of Biomedical Sciences, Houston, TX, USA

**Keywords:** CXCR4, DLBCL, BCL2, Myc, TP53 mutation

## Abstract

Abnormal expression of the chemokine receptor CXCR4 plays an essential role in tumor cell dissemination and disease progression. However, the significance of CXCR4 overexpression in *de novo* diffuse large B cell lymphoma (DLBCL) is unknown. In 743 patients with *de novo* diffuse large B cell lymphoma (DLBCL) who received standard Rituximab-CHOP immunochemotherapy, we assessed the expression of CXCR4 and dissected its prognostic significance in various DLBCL subsets. Our results showed that CXCR4^+^ patients was associated with male, bulky tumor, high Ki-67 index, activated B-cell-like (ABC) subtype, and Myc, Bcl-2 or p53 overexpression. Moreover, CXCR4^+^ was an independent factor predicting poorer progression-free survival in germinal-center B-cell-like (GCB)-DLBCL, but not in ABC-DLBCL; and in patients with an IPI of ≤2, but not in those with an IPI>2. The lack of prognostic significance of CXCR4 in ABC-DLBCL was likely due to the activation of p53 tumor suppressor attenuating CXCR4 signaling. Furthermore, concurrent CXCR4^+^ and *BCL2* translocation showed dismal outcomes resembling but independent of *MYC/BCL2* double-hit DLBCL. Gene expression profiling suggested that alterations in the tumor microenvironment and immune responses, increased tumor proliferation and survival, and the dissemination of CXCR4^+^ tumor cells to distant organs or tissues were underlying molecular mechanisms responsible for the CXCR4^+^ associated poor prognosis.

## INTRODUCTION

CXCR4 (CD184) is a chemokine receptor specific for CXCL12. The CXCL12/CXCR4 axis is critical to the retention of B-cell precursors in bone marrow (BM), homing of B lymphocytes to lymph nodes, and infiltration of T-cells and other immune cells expressing CXCR4 [1]. Signaling molecules, physiological stimuli, and co-translational modifications control the expression, oligomerization, internalization, and degradation of CXCR4. CD63, interleukin 21, hypoxia-inducible factor 1 alpha, nuclear factor-kappa B (NF-κB), CREB3, PAX3-FKHR, Wnt, Notch, and PI3K/Akt pathways positively regulate CXCR4 levels. In contrast, p53 [2], tumor necrosis factor-alpha (TNF-α), interferon-gamma, and ubiquitination modification negatively regulate CXCR4 levels [3-6]. Activated CXCL12/CXCR4 in turn activates signaling cascades such as PI3K/Akt, mitogen-activated protein kinase (MAPK), integrin, tyrosine kinases, and G-proteins [3,4].

Abnormal CXCR4 surface expression in solid tumors, has been shown to be responsible for their metastasis to particular organs with high CXCL12 levels (e.g., lymph nodes, bones, and BM) [3,7,8], and have prognostic significance for disease progression in breast, colorectal, and renal cancers, and hepatocellular carcinoma [3,9,10]. In leukemia, CXCR4 expression conferred leukemic blasts with a higher capacity to seed into BM niches, thereby protecting leukemic cells from chemotherapy-induced apoptosis, and was correlated with shorter disease-free survival [3,11-15]. Conversely, neutralizing the interactions of CXCL12/CXCR4 disrupted metastasis, induced apoptosis, and increased chemosensitivity in solid cancers and leukemia [7,16-18].

Diffuse large B cell lymphoma (DLBCL) is the most common type of non-Hodgkin lymphoma among adults. DLBCL typically presents as a nodal or extranodal mass with rapid tumor growth. Extranodal DLBCL (primary sites are outside the lymphatic system) accounts for 30-40% of DLBCL. Approximately 70% of DLBCLs have at least one and 30% have multiple extranodal involvements [19,20]. With the standard immunochemotherapy regimen consisting of rituximab, cyclophosphamide, doxorubicin, vincristine, and prednisone (R-CHOP), approximately one-third of DLBCL patients develop relapsed/refractory disease [21]. Gene expression profiling (GEP) divides DLBCL into two main subtypes according to cell-of-origin gene signatures: germinal center B-cell-like (GCB), arising from the germinal center (GC) compartment, and activated B-cell-like (ABC), arising from post-GC plasmablastic cells [22]. During the development of mature B-cells, CXCR4 is expressed at higher levels in centroblasts localized in the CXCL12-rich dark zone than in centrocytes in the light zone of the GC. CXCR4 is also upregulated during plasma cell differentiation and expressed in memory B-cells [23-27].

The prognostic significance of CXCR4 expression in lymphoma, which has different CXCL12 gradients at the primary sites compared to other types of cancers [28], has not been well studied. Moreover, it is unknown whether the use of a CXCL12/CXCR4 antagonist in nodal DLBCL will result in lymphoma cell mobilization and increased spreading [8,29-32]. Very recently, CXCR4 expression was correlated to disease progression in 12 cases of primary testicular DLBCL [33] and poor survival of 94 DLBCL cases [34]. In 20 patients with non-Hodgkin lymphomas, a significant decrease in CXCR4 mRNA expression in the BM after treatment correlated with a significantly lower risk of death [35]. In this study, we assessed the surface expression of CXCR4 using immunohistochemistry (IHC) in 743 patients with *de novo* DLBCL, compared the gene expression profiles and protein expression of biomarkers between CXCR4^+^ and CXCR4^−^ DLBCLs, and evaluated the prognostic value of CXCR4 expression. We also tested the effect of the high-affinity CXCL12/CXCR4 inhibitor BTK140 (4F-benzoyl-TN14003) on DLBCL cells *in vitro*, which not only inhibits CXCL12/CXCR4 mediated adhesion and migration [36], overcomes stromal cells-mediated chemoresistance, but also has direct cytotoxic activities in non-Hodgkin lymphoma cell lines, leukemic and multiple myeloma cells in a CXCR4-dependent and dose-dependent manner [29,37].

## RESULTS

### CXCR4 and CXCL12 expression

**Figure 1:**
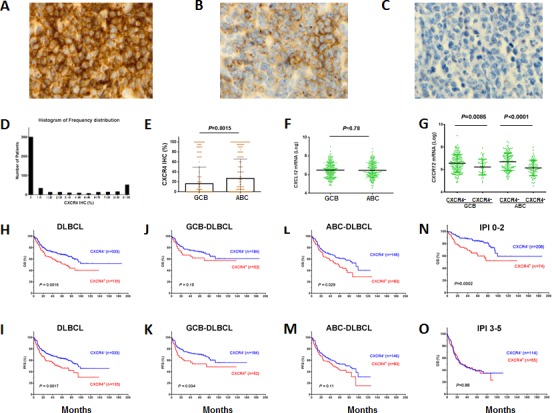
Expression and prognostic significance of CXCR4 in DLBCL (A-C) Representative CXCR4 immunohistochemistry staining (showing 100%, 60%, 0% CXCR4 cell surface expression in DLBCL cells). (D) Histogram of CXCR4 expression frequency distribution in the DLBCL study cohort. X-axis, percentage of immunopositive cells in tumors; Y-axis, numbers of DLBCL patients. (E-F) ABC-DLBCL compared to GCB-DLBCL had increased CXCR4 cell surface expression, but did not differ in *CXCL12* mRNA significantly. (G) CXCR4 cell surface expression correlated with decreased *CXCL12* mRNA levels, both in GCB- and ABC-DLBCL. (H-I) CXCR4 expression correlated with significantly poorer OS and PFS in the overall DLBCL cohort. (J-K) CXCR4 expression correlated with significantly poorer PFS (but not OS) in GCB-DLBCL. (L-M) CXCR4 expression correlated with significantly poorer OS (but not PFS) in ABC-DLBCL. (N-O) CXCR4 expression correlated with significantly poorer survival in DLBCL patients with a low IPI, but not in DLBCL patients with a high IPI.

IHC results (representative positive and negative staining was shown in Figures 1A-C) indicated that in most of the DLBCLs, CXCR4 surface expression level was low (Figure 1D, Supplemental Figures 1A-B). The mean expression level in the 468 DLBCLs of the training set was 20% of tumor cells positive for CXCR4 cell surface expression, which was used as the cutoff for CXCR4 overexpression (CXCR4^+^). Using this cutoff (≥20%), we found that 28.8% of the samples in the training cohort were CXCR4^+^.

CXCR4 cell surface expression and mRNA levels were higher in the ABC than GCB subtype, whereas *CXCL12* mRNA levels did not differ significantly between the two groups (Figures 1E-F, Supplemental Figure 1C). CXCR4 expression detected via IHC was significantly correlated with CXCR4 mRNA levels (*P* < .0001, Supplemental Figure 1D), and intriguingly, significantly correlated with lower *CXCL12* mRNA levels (Figure 1G).

### Clinicopathologic features of patients with CXCR4 expression

Clinically, CXCR4^+^ group had higher proportion of male patients and patients with bulky tumors than the CXCR4^−^ group, and tended to have higher frequency of >1 extranodal involvement (*P*= .089) (Table 1). Pathologically, CXCR4^+^ GCB-DLBCLs compared to CXCR4^−^ GCB-DLBCLs more frequently had a high Ki-67 index, *TP53* mutations, Myc overexpression and less frequently expressed BLIMP-1 or nuclear RelB. In comparison, CXCR4^+^ ABC-DLBCLs compared to CXCR4^−^ ABC-DLBCLs had a higher percentage of patients with a high Ki-67 index, p53, Myc, Bcl-2, PI3K expression and lower occurrence of *BCL6* translocations and nuclear p50 expression (Table 2).

**Table 1:** Clinical features of patients with CXCR4^+^ and CXCR4^−^ expression in overall, GCB-DLBCL and ABC-DLBCL

	DLBCL			GCB-DLBCL			ABC-DLBCL		
Variables	CXCR4^+^ N(%)	CXCR4^−^ N(%)	*P*	CXCR4^+^ N(%)	CXCR4^−^ N(%)	*P*	CXCR4^+^ N(%)	CXCR4^−^ N(%)	*P*
**Age, years**									
< 60	51(37.8)	147(44.1)	.21	28(53.8)	93(50.5)	.67	23(27.7)	53(36.3)	.18
≥ 60	84(62.2)	18655.9%		24(46.2)	91(49.5)		60(72.3)	93(63.7)	
**Gender**									
F	40(29.6)	148(44.4)	**.0031**	18(34.6)	78(42.4)	.31	22(26.5)	69(47.3)	**.002**
M	95(70.4)	185(55.6)		34(65.4)	106(57.6)		61(73.5)	77(52.7)	
**Stage**									
I - II	56(42.4)	155(48.3)	.26	23(45.1)	98(55.7)	.18	33(40.7)	55(39)	.80
III - IV	76(57.6)	166(51.7)		28(54.9)	78(44.3)		48(59.3)	86(61.0)	
**B-symptoms**									
No	84(65.6)	206(64.8)	.87	34(70.8)	123(70.3)	.94	50(62.5)	80(57.6)	.47
Yes	44(34.4)	112(35.2)		14(29.2)	52(29.7)		30(37.5)	59(42.4)	
**LDH level**									
Normal	45(36.6)	122(39.9)	.53	19(42.2)	72(42.6)	.96	26(33.3)	49(36.8)	.61
Elevated	78(63.4)	184(60.1)		26(57.8)	97(57.4)		52(66.7)	84(63.2)	
**Number of extranodal sites**									
0 - 1	94(73.3)	251(79.9)	**.089**	35(72.9)	140(80)	.29	59(71.1)	108(77.7)	.27
≥ 2	37(26.7)	66(20.1)		13(27.1)	35(20)		24(28.9)	31(22.3)	
**ECOG Performance status**									
0 - 1	104(85.2)	243(82.9)	.56	39(90.7)	134(83.8)	.25	65(82.3)	106(81.5)	.89
≥ 2	18(14.8)	50(17.1)		4(9.3)	26(16.3)		14(17.7)	24(18.5)	
**Size of largest tumor**									
< 5cm	55(49.1)	151(62.1)	**.02**	22(75.9)	82(60.3)	.11	33(45.2)	67(65)	**.0088**
≥ 5cm	57(50.9)	92(37.9)		7(24.1)	54(39.7)		40(54.8)	36(35)	
**IPI score**									
0 - 2	74(57.4)	208(64.4)	.16	31(64.6)	125(70.6)	.42	43(53.1)	80(56.3)	.64
3 - 5	55(42.6)	115(35.6)		17(35.4)	52(29.4)		38(46.9)	62(43.7)	
**Therapy response**									
CR	102(75.6)	261(78.4)	.51	35(67.3)	148(80.4)	**.045**	67(80.7)	111(76)	.41
PR	13	45		5	21		8	24	
SD	8	11		5	7		3	4	
PD	12	16		7	8		5	7	
**COO**									
GCB	52(38.5)	184(55.8)	**.0008**						
ABC	83(61.5)	146(44.2)							

Abbreviations: LDH, lactate dehydrogenase; IPI, International Prognostic Index; CR, complete remission; PR, partial response; SD, stable disease; PD, progressive disease; COO, cell-of-origin. For therapy response, we calculated *P* values as CR vs other responses. Few clinical features of certain cases were not available.

### CXCR4 expression was associated with significantly poorer survival

CXCR4^+^ DLBCL patients had significantly poorer overall survival (OS) (*P*= .0016) and progression-free survival (PFS) (*P*= .0017) in the study group (Figures 1H-I). When examined in the GCB and ABC subtypes, the adverse impact was significant for the PFS of patients with CXCR4^+^ GCB-DLBCL (Figure 1K), and the OS of patients with CXCR4^+^ ABC-DLBCL (Figure 1L). Further multivariate analysis adjusting for clinical factors of the study cohort indicated that CXCR4^+^ remained as an independent prognostic factor for significantly poorer OS (*P*= .02) and PFS (*P*= .03) in the overall DLBCL; However, only in GCB- but not in ABC-DLBCL patients, CXCR4^+^ expression was as an independent prognostic factor for poorer PFS (*P*= .025). Interestingly, only in patients with ABC-DLBCL was CXCR4^+^ expression associated with wide-type (WT) p53 expression in the study cohort (Supplemental Figure 1E, Table 2).

**Table 2:** Pathological features of patients with CXCR4^+^ and CXCR4^−^ expression in overall, GCB-DLBCL and ABC-DLBCL

	DLBCL			GCB-DLBCL			ABC-DLBCL		
Variables	CXCR4^+^ N(%)	CXCR4^−^ N(%)	*P*	CXCR4^+^ N(%)	CXCR4^−^ N(%)	*P*	CXCR4^+^ N(%)	CXCR4^−^ N(%)	*P*
**Ki-67 index**									
< 70%	28(20.7)	137(42.3)	**< .0001**	11(21.2)	79(44.6)	**.002**	17(20.5)	57(39)	**.005**
≥ 70%	107(79.3)	187(57.7)		41(78.8)	98(55.4)		66(79.5)	89(61)	
***TP53* mutations**									
No	94(74)	241(80.9)	**.07**	29(61.7)	133(78.7)	**.017**	65(81.3)	105(83.3)	.70
Yes	33(26)	57(19.1)		18(38.3)	36(21.3)		15(18.8)	21(16.7)	
***MYC* translocation**									
No	83(85.6)	198(91.7)	.11	25(80.6)	102(87.9)	.37	58(87.9)	95(96)	**.067**
Yes	14(14.4)	18(8.3)		6(19.4)	14(12.1)		8(12.1)	4(4)	
***BCL2* translocation**									
No	101(83.5)	227(82.2)	**.89**	29(67.4)	107(70.4)	.71	72(92.3)	119(97.5)	**.16**
Yes	20(16.5)	49(17.8)		14(32.6)	45(29.6)		6(7.7)	3(2.5)	
***BCL6* translocation**									
No	73(83)	150(64.4)	**.0013**	32(82.1)	95(73.1)	.26	41(69.5)	54(52.9)	**.04**
Yes	15(17)	83(35.6)		7(17.9)	35(26.9)		18(30.5)	48(47.1)	
**p53 overexpression**									
< 20%	71(57.3)	195(67.7)	**.044**	29(63)	104(63.8)	1.00	42(53.8)	91(72.8)	**.0065**
≥ 20%	53(42.7)	93(32.3)		17(37)	59(36.2)		36(46.2)	34(27.2)	
**Myc overexpression**									
< 70%	73(57.9)	221(72.5)	**.0044**	31(60.8)	126(78.3)	**.017**	42(56)	93(65)	.24
≥ 70%	53(42.1)	84(27.5)		20(39.2)	35(21.7)		33(44)	50(35)	
**Bcl-2 overexpression**								
< 70%	48(35.8)	175(53.7)	**.0007**	24(47.1)	108(60)	**.11**	24(28.9)	65(45.1)	**.017**
≥ 70%	86(64.2)	151(46.3)		27(52.9)	72(40)		59(71.1)	79(54.9)	
**GCET1 overexpression**								
< 50%	84(63.2)	219(67.6)	.38	21(40.4)	89(49.7)	.27	63(77.8)	129(89.6)	**.019**
≥ 50%	49(36.8)	105(32.4)		31(59.6)	90(50.3)		18(22.2)	15(10.4)	
**FOXP1 overexpression**								
< 60%	33(24.4)	151(46.3)	**< .0001**	26(50)	118(65.6)	**.05**	7(8.4)	33(22.6)	**.0065**
≥ 60%	102(75.6)	175(53.7)		26(50)	62(34.4)		76(91.6)	113(77.4)	
**MUM1 overexpression**								
< 30%	43(31.9)	181(55.7)	**< .0001**	34(65.4)	137(76.1)	**.15**	9(10.8)	44(30.3)	**.006**
≥ 30%	92(68.1)	144(44.3)		18(34.6)	43(23.9)		74(89.2)	101(69.7)	
**PI3K overexpression**								
< 70%	80(61.5)	233(73.7)	**.012**	36(73.5)	130(73.4)	1.00	44(54.3)	103(74.6)	**.0028**
≥ 70%	50(38.5)	83(26.3)		1326.5%	47(26.6)		37(45.7)	35(25.4)	
**BLIMP-1 expression**									
< 10%	97(75.2)	233(72.6)	.64	47(95.9)	144(80.9)	**.008**	50(62.5)	88(62)	1.00
≥ 10%	32(24.8)	88(27.4)		2(4.1)	34(19.1)		30(37.5)	54(38)	
**p50**									
Negative	74(57.4)	135(44.4)	**.016**	34(66.7)	93(54.7)	.15	40(51.3)	42(31.3)	**.0054**
Positive	55(42.6)	169(55.6)		17(33.3)	77(45.3)		38(48.7)	92(68.7)	
**p52**									
Negative	100(77.5)	210(67.7)	**.05**	38(77.6)	114(67.1)	**.21**	62(77.5)	96(68.6)	.17
Positive	29(22.5)	100(32.3)		11(22.4)	56(32.9)		18(22.5)	44(31.4)	
**p65**									
Negative	60(45.8)	129(40.7)	.34	22(44)	65(37.8)	.51	38(46.9)	61(42.7)	.58
Positive	71(54.2)	188(59.3)		28(56)	107(62.2)		43(53.1)	82(57.3)	
**RelB**									
Negative	117(92.1)	253(82.4)	**.01**	46(95.8)	143(83.6)	**.03**	71(89.9)	109(80.7)	**.08**
Positive	10(7.9)	54(17.6)		2(4.2)	28(16.4)		8(10.1)	26(19.3)	
**c-Rel**									
Negative	89(68.5)	208(69.3)	.91	31(62)	121(72.9)	.16	58(72.5)	87(64.9)	.29
Positive	41(31.5)	92(30.7)		19(38)	45(27.1)		22(27.5)	47(35.1)	

International Prognostic Index (IPI) score appeared to be a determinant of CXCR4 prognostic significance. Only in patients with an IPI ≤2 was CXCR4^+^ expression associated with significantly poorer OS (Figure 1N) and PFS (*P*= .0002). In patients with an IPI >2, CXCR4^+^ expression did not have distinguishable prognostic significance (OS, *P*= .88, Figure 1O; PFS, *P*= .91).

**Figure 2:**
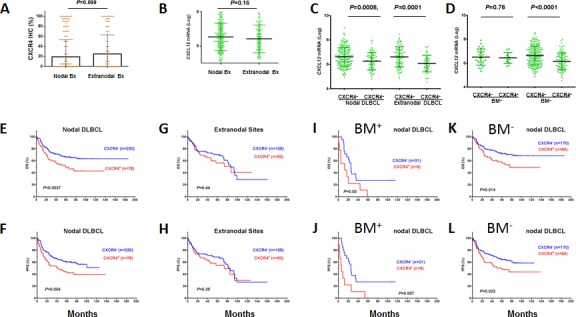
Expression and prognostic significance of CXCR4 in nodal and extranodal DLBCL (A-B) CXCR4 cell surface and *CXCL12* mRNA expression levels in nodal and extranodal DLBCL. (C) CXCR4 cell surface expression correlated with decreased *CXCL12* mRNA levels, both in nodal and extranodal DLBCL. (D) CXCR4 cell surface expression correlated with decreased *CXCL12* mRNA levels in DLBCL patients without bone marrow (BM) involvement. (E-F) CXCR4 expression correlated with significantly poorer OS and PFS in the nodal DLBCL. (G-H) CXCR4 expression in extranodal sites did not correlate with survival significantly in DLBCL. (I-L) the prognostic significance of CXCR4 expression was independent of BM involvement.

Since CXCL12 gradients differ in lymph nodes, BM, and other tissues affecting chemotaxis, we analyzed the prognostic impact of CXCR4 expression in lymph nodes and extranodal sites separately (Figures 2A-B showed CXCR4 cell surface expression and *CXCL12* mRNA levels in nodal vs primary extranodal patients). Although CXCR4 cell surface expression invariably correlated with lower *CXCL12* mRNA levels in both nodal and extranodal sites (Figure 2C), CXCR4^+^ expression correlated with significantly poorer OS and PFS only in nodal DLBCLs (Figures 2E-H) regardless of extranodal involvement status (Supplemental Figure 1F). In contrast, CXCR4 surface expression was negatively correlated with *CXCL12* mRNA levels only in patients without BM involvement (Figure 2D). However, the prognostic significance of CXCR4 in nodal DLBCL was demonstrated in both groups either with or without BM involvement at diagnosis (Figures 2I-L). Together, these data suggested that the prognostic significance of CXCR4 expression is independent of BM or extranodal involvement, and reduction of *CXCL12* mRNA levels in the primary sites.

### Association and synergy among CXCR4, Bcl-2, and Myc expression in GCB-DLBCL

**Figure 3:**
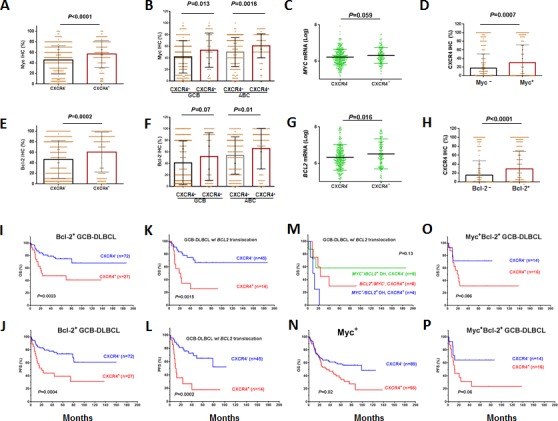
Association of CXCR4 expression with Myc/Bcl-2 expression and the synergism of prognostic significance in DLBCL (A-D) Association between CXCR4 and Myc expression levels. (E-H) Association between CXCR4 and Bcl-2 expression levels. (I-J) CXCR4 expression synergized with Bcl-2 expression in GCB-DLBCL. (K-L) CXCR4 expression synergized with *BCL2* translocation in GCB-DLBCL. (M) The synergism between *BCL2* translocation and CXCR4 expression was independent of double-hit MYC/*BCL2* translocations. (N) CXCR4 expression synergized with Myc expression in DLBCL. (O-P) the prognostic significance of CXCR4 in GCB-DLBCL patients with concurrent Myc/Bcl-2 expression.

CXCR4, Myc and Bcl-2 expression showed association in both the GCB and ABC subtypes (Figures 3A-H). Myc and Bcl-2 expression, and *MYC* and *BCL2* translocation have been correlated with poor clinical outcomes [38-40]. We therefore assessed the dependency and synergism among the prognostic impact of CXCR4, Myc, and Bcl-2 expression.

Although the inverse correlation between CXCR4 surface expression and *CXCL12* mRNA levels was independent of Bcl-2/Myc expression status (Supplemental Figures 3A-D), CXCR4^+^ expression correlated with significantly poorer survival in patients with Bcl-2^+^ GCB-DLBCL (Figures 3I-J) or Bcl-2^−^ ABC-DLBCL, but not in patients with Bcl-2^−^ GCB-DLBCL or Bcl-2^+^ ABC-DLBCL (Supplemental Figure 2). Within the GCB-DLBCL group, in which CXCR4^+^ and *BCL2* translocations are prognostic [40], CXCR4 expression showed remarkable synergism with *BCL2* translocations (Figures 3K-L), in a manner no less significant than the synergism between *MYC* and *BCL2* translocations (Figure 3M).

Similarly, CXCR4 expression was synergistic with Myc overexpression (Figure 3N); however, when the group was classified into GCB and ABC subtypes, this result did not remain statistically significant (Supplemental Figures 1G-J).

Among Myc^+^/Bcl-2^+^ patients, CXCR4 expression had an additive adverse impact in patients with GCB-DLBCL (Figures 3O-P, *P*= .08 for OS and *P*= .06 for PFS), but this impact was not statistically significant in patients with ABC-DLBCL (Supplemental Figures 1K-L).

### Association of CXCR4 expression with TP53 mutations in GCB-DLBCL

**Figure 4:**
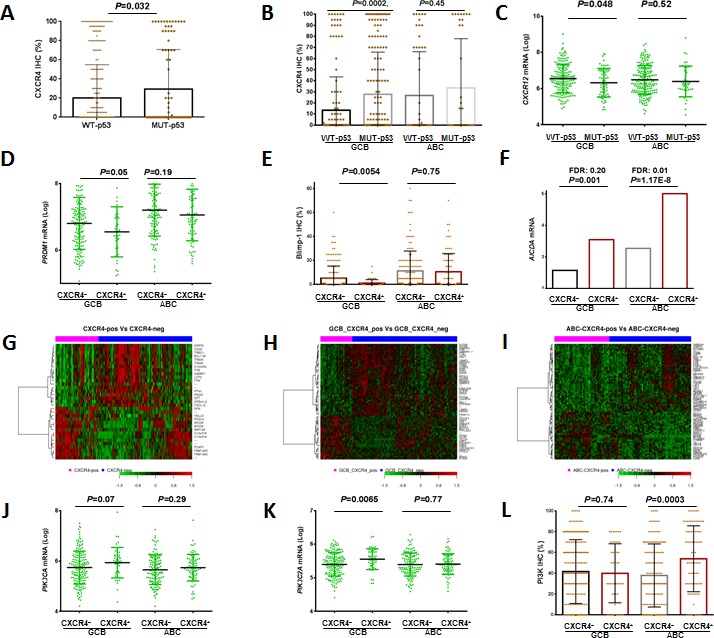
Regulation of and signaling pathways related to CXCR4 expression (A-C) p53 mutations were associated increased CXCR4 and decreased *CXCL12* mRNA expression, especially in GCB-DLBCL. (D-E) CXCR4 expression was associated with decreased *PRDM1* mRNA and BLIMP-1 expression in GCB-DLBCL. (F) CXCR4 expression was associated with increased *AICDA* mRNA expression both in GCB- and ABC-DLBCL. (G-H) Heatmaps and differentially expressed genes between CXCR4^+^ and CXCR4^−^ patients in the overall DLBCL, GCB-DLBCL and ABC-DLBCL cohorts. (J-K) CXCR4 expression was associated with increased *PI3K* mRNA expression in GCB-DLBCL, and increased protein expression in ABC-DLBCL.

In CXCR4^+^ GCB-DLBCL, the frequency of TP53 mutations (which correlated with poor clinical outcomes [41]) was much higher than in CXCR4^−^ GCB-DLBCL (38.3% *vs* 21.3%, *P*= .017, Table 2). However, the adverse impact of CXCR4 expression was independent of TP53 mutations (Supplemental Figure 3G-H). Conversely, patients with mutated (MUT)-p53 expressed higher CXCR4 levels and lower *CXCL12* mRNA levels than patients with WT-p53, with significant *P* values within the GCB subtype (Figures 4A-C).

**Table 3:** Multivariate survival analysis [Gender, IPI (age, stage, LDH, ECOG, number of extranodal sites), tumor size, B-symptoms, CXCR4, Myc, Bcl-2, and p53 expression]

	OS			PFS		
Variables	HR	95% CI	*P*	HR	95% CI	*P*
**Overall DLBCL**						
**IPI > 2**	2.33	1.70-3.21	**< .0001**	2.13	1.58-2.88	**< .0001**
**CXCR4^+^**	1.12	.79-1.69	.27	1. 56	1.13-2.46	**.008**
**Myc^+^**	2.21	1.55-3.14	**< .0001**	2.11	1.51-2.94	**< .0001**
**Bcl-2^+^**	1.48	1.04-2.10	.028	1.27	.94-2.10	.028
**TP53 mutations**	1.66	1.11-2.51	**.013**	1. 07	1.17-2.46	**.005**
**p53^+^**	1.21	.84-1.75	.30	1.17	.83-1.66	.38
**Female**	.88	.62-1.25	.48	.93	.67-1.30	.68
**>5cm tumor**	1.24	.91-1.69	.18	1.16	.87-1.55	.33
**B-symptoms**	1.36	.99-1.87	.06	1.32	.98-1.79	.07
**GCB-DLBCL**						
**IPI > 2**	3.66	2.09-6.38	**< .0001**	3.95	2.40-6.50	**< .0001**
**CXCR4^+^**	1.35	.74-2.46	.33	1.75	1.02-3.01	**.04**
**Myc^+^**	2.83	1.57-5.10	**.0001**	2.39	1.43-4.00	**.0001**
**Bcl-2^+^**	2.00	.96-3.90	.06	1.73	.94-3.19	.08
**TP53 mutations**	1.48	.79-2.77	.21	1.74	1.03-2.94	**.04**
**Female**	.82	.47-1.43	.49	.90	.54-1.50	.69
**>5cm tumor**	1.59	.92-2.74	.097	1.53	.93-2.53	.096
**B-symptoms**	1.72	.99-3.00	**.05**	1.52	.90-2.54	.12
**ABC-DLBCL**						
**IPI > 2**	2.85	1.76-4.64	**< .0001**	2.33	1.47-3.68	**< .0001**
**CXCR4^+^**	1. 32	.77-1.25	.32	1. 65	.86-2.80	.14
**Myc^+^**	1.47	.90-2.38	.11	1.48	.94-2.33	.09
**Bcl-2^+^**	2.43	1.33-4.45	**.004**	2.21	1.26-3.88	**.006**
**TP53 mutations**	2.00	1.07-3.71	**.029**	1.80	.95-3.36	.06
**Female**	.70	.42-1.18	.18	.72	.43-1.31	.21
**>5cm tumor**	1.12	.67-1.88	.66	.95	.56-1.60	.83
**B-symptoms**	1.76	1.05-2.93	**.031**	1.49	.89-2.52	.13

**Abbreviations**: OS, overall survival; PFS, progression-free survival; HR, hazard ratio; CI, confidence interval; IPI, International Prognostic Index.

Blimp-1 was another tumor suppressor that was significantly downregulated in CXCR4^+^ GCB-DLBCL (Figures 4D-E), and this downregulation may also contribute to the prognostic impact of CXCR4 overexpression.

### Multivariate survival analysis of CXCR4, Myc, Bcl-2, and TP53 mutations

Since CXCR4 expression was associated with Myc/Bcl-2 expression and TP53 mutations, all of which are adverse prognostic factors, multivariate survival analysis of the pathological factors (including CXCR4^+^, Myc^+^, Bcl-2^+^ and TP53 mutations) and the clinical parameters (including IPI, gender, tumor size, and B symptoms) was performed, which indicated CXCR4 was an independent prognostic factor for disease progression (hazard ratio 1.56, 95% confidence interval of rate 1.13-2.46, *P*= .008. Table 3).

**Table 4:** Up- and down-regulated genes in patients with CXCR4^+^ and CXCR4^−^ expression in overall DLBCL (false discovery rate [FDR] threshold: .01; *P* value cutoff: .000098; fold change cutoff: 1.41), GCB-DLBCL (FDR threshold: .10, *P* value cutoff: .000156), and ABC-DLBCL (FDR threshold: .05, *P* value cutoff: .000109)

CXCR4^+^ *vs.* CXCR4^−^						
	DLBCL		GCB-DLBCL		ABC-DLBCL	
	Upregulated	Downregulated	Upregulated	Downregulated	Upregulated	Downregulated
**Signaling, ion channels**	*CXCR4, SFN, TMC5*	*DUSP4, GIMAP7, CSF2RB, CECR1*	*CNR1, TMC5, STIM2, DOK5, PTHLH*	*GABBR1, UBD, DUSP4, FYB, PTPN6, LAMP1, NTNG2, RASA3*	*YTHDF2*	*FYN, FYB, PBXIP1, MS4A6A, PLCD1, ACVRL1, ADAP2*
**Cytokine, chemokine**		*CXCL12, CCL2*				*CXCL12, TNFSF12*
**Immune responses, inflammation**	*IRF4, TCF4*	*HLA-DQA1/HLA-DQA2, HLA-DRB1/HLA-DRB4, TRBC1, GIMAP1, FYN, FYB, LCP2, CD3E, SIRPG, SAMHD1, C3, LAT*	*POU2AF1*	*LCP2, LILRB2, SAMHD1, HLA-E*		*TRBC1, STAT4, C2, LST1*
**DNA recombination, mitotic regulation**	*AICDA, HELLS, ZWINT*		*MLLT11, SGOL2, FRY*		*AICDA, HELLS, ANLN, CCDC52, CDK2, CEP152, RAD54B*	
**Transcription regulation**	*FOXP1, CDCA7, AFF3*	*BCL11B*		*MKL1*	*MYEF2*	*BCL11B, TXNIP, CTBP2*
**mRNA editing, translation**		*ADARB1*	*HRSP12*		*FARSA*	*SAMD4A*
**Metabolism**	*TMEM97, FAM72A*		*PTPLAD1*	*RGL1, CYP46A1*	*HILPDA, CYP51A1, MOCOS*	*SULT1A3/SULT1A4, SULT1A2*
**Transport, protein modification, folding, chaperone, degradation**	*DNAJC6*	*RTN1, PADI2*	*TCL1A, DNAJC6, AP4S1*	*RTN1, APOL3, PARP14, PSAP, NAGA, CYLD*	*SUGT1*	*FOLR2, SLC46A1, SLC7A6OS, MARCH2*
**Cell adhesion, cytoskeleton, collagen, extracelluar matrix**		*DPT, EPB41L2, ITGB2, UTRN SH3KBP1*	*EML6, STK33*	*ITGB2, MYO1F*	*FLJ23834, TTC30A, NPHP1*	*MFAP4, ITGAL, EVL, CD6, FGD3, SIGLEC7*
**Differentiation, development**		*ITM2A, SLAMF8*		*SLFN5*	*FIGNL1, DAZAP1*	*TMEM2*
**Apoptosis, autophage**	*PEG10*	*MAF, RASSF4*		*RASSF4, GIMAP5*	*PEG10, PIM2, BECN1*	*TBRG1*
**IncRNA genes, unknown function**	*RNF183, P704P, C13orf18, FAM129C, KLHL23*	*C16orf54, EPSTI1*	*TCL6, ST7OT4, LOC100131683*	*C6orf204, FAM105A*	*DPY19L2P2, C9orf40, CCDC117*	*LOC100131096, LOC400236, LOC646014, GDPD3, N4BP2L1*

However, when dissected in the GCB and ABC subtypes, the independent prognostic significance of CXCR4^+^ was limited to GCB-DLBCL (*P*= .04 for PFS); in ABC-DLBCL, Myc and Bcl-2 overexpression and TP53 mutations but not CXCR4 expression, independently predicted poorer survival (Table 3).

We validated the prognostic significance of CXCR4 in an independent DLBCL cohort (n=275) and confirmed that the prognostic significance of CXCR4 was most common in patients with an IPI ≤2, depended on Bcl-2 overexpression in GCB-DLBCL, and had synergy with Myc expression (Supplemental Figure 4).

### Differentially expressed genes in CXCR4^+^ versus CXCR4^−^ DLBCL patients

We compared the GEP of CXCR4^+^ and CXCR4^−^ DLBCLs, and found that 447 genes were significantly differentially expressed with a false discovery rate (FDR) threshold .01 and a fold change cutoff of over 1.41 (Table 4). Likely owing to the significantly reduced *CXCL12* expression, which facilitates T cell infiltration and trafficking, the GEP of patients with CXCR4^+^ DLBCL revealed remarkably lower expression of T-cell and innate immune response biomarkers (MHC class II molecules *HLA-DQA1/HLA-DQA2*, *HLA-DRB1/HLA-DRB4*, *TRBC1*, *GIMAP1*, *FYN*, *FYB*, *LCP2*, *CD3E*, *SIRPG*, *C3*, *LAT*, *MAF*, and *SAMHD1* involved in antigen presentation and T cell signaling) indicating worse prognosis [42], and cell adhesion genes. In addition, CXCR4 gene signatures also included upregulated survival genes and downregulated pro-apoptosis genes in CXCR4^+^ tumor cells. Upregulated genes included *SFN* (2.57 fold) which stimulates the Akt/mTOR pathway, *HELLS* which is involved in lymphoid cell survival (1.55 fold), Myc-responsive gene *CDCA7* which contributes to the Myc-mediated tumorigenesis (1.51 fold), oncogenic transcription factor *AFF3* (1.45 fold), *FAM72A* which regulates cell growth (1.44 fold), and antipoptotic *PEG10* (1.85 fold). In contrast, pro-apoptotic *RASSF4* was downregulated (1.42 fold). *AICDA*, encoding activation-induced cytidine deaminase which mediates somatic hypermutation and class-switch recombination, was upregulated by 2.92 fold in patients with CXCR4^+^ DLBCL (3.32 fold in ABC-DLBCL and 2.12 fold in GCB-DLBCL, Figures 4F-G). Furthermore, these signatures largely overlapped the differentially expressed genes (DEGs) identified between CXCR4^+^ and CXCR4^−^ DLBCL patients with an IPI ≤2 (Supplemental Figure 3I and Table 5), whereas no DEGs were identified between CXCR4^+^ and CXCR4^−^ DLBCLs with an IPI >2.

**Table 5:** Up- and down-regulated genes in DLBCL patients with CXCR4^+^ and CXCR4^−^ expression and an IPI≤2 (false discovery rate threshold: .01; *P* value cutoff: .000058; fold change cutoff: 1.57)

	CXCR4^+^ *vs.* CXCR4^−^ DLBCL with an IPI ≤2	
	Upregulated	Downregulated
**Signaling, ion channels**		*GIMAP2*
**Cytokine, chemokine**		*CXCL12*
**Immune responses, inflammation**		*SIRPG, TRBC1, LAT, FYB, FYN, LCP2, GVIN1, HLA-DQA1/HLA-DQA2, HLA-DRB1/HLA-DRB4,*
**DNA recombination, mitotic regulation**	*AICDA, HELLS, ZWINT*	
**Transcription regulation**	*CDCA7, AFF3*	*BCL11B, TXNIP*
**mRNA editing, translation**		*ADARB1*
**Metabolism**	*FAM72A*	
**Transport, protein modification, folding, chaperone, degradation**	*AP1S3*	*SPNS1, APOC1, RTN1*
**Cell adhesion, cytoskeleton, collagen, extracelluar matrix**		
**Differentiation, development**		*ITM2A, SLAMF8*
**Apoptosis, autophage**	*RASSF6, PEG10*	*MAF, RASSF4*
**IncRNA genes, unknown function**	*C13orf18, FAM129C, RNF183, KLHL23, DPY19L2P2*	

GCB-DLBCL and ABC-DLBCL have distinct molecular programs, therefore, CXCR4 expression signatures were further identified in the GCB and ABC subsets separately (Figures 4H-I and Table 4). The immunosuppressive, proliferative, and antiapoptotic CXCR4 signatures were observed in both GCB and ABC subtypes. In GCB-DLBCL, downregulation of *FYB, LCP2, LILRB2, SAMHD1,* and *HLA-E*, suggested decreased adaptive and innate immune responses. In ABC-DLBCL, downregulation of *FYN, FYB, TRBC1, STAT4, C2*, and *LST1*, suggested decreased adaptive immune responses. In ABC-DLBCL, the proliferation and antiapoptotic CXCR4 signatures were remarkable, such as upregulation of genes involved in the cell cycle progression, mitosis, translation, metabolism and antiapoptosis (including *CDK2*, *HELLS*, *CCDC52*, *FARSA*, hypoxia-inducible lipid-droplet-associated protein [*HILPDA*], *PEG10, PIM2*, and *BECN1*), and downregulation of the mTORC1 inhibitor *TXNIP*, the tumor suppressors *BCL11B* and *TBRG1*. The involvement of the Myc and TP53 pathways in the CXCR4 signaling was suggested by the upregulation of *PIM2*, which increases Myc stability and transcriptional activity, and the downregulation of *BCL11B* and *TBRG1*, which activate p53.

Many genes were differentially regulated in GCB and ABC subtypes, including the ones involved in the PI3K pathway (Figures 4J-L), MAPK signaling, NF-κB and angiogenesis (Table 4). In GCB-DLBCL, positive regulation of the MAPK pathway by CXCR4 expression was suggested by the upregulation of *DOK5*, *PTHLH* (which transports calcium), and *STIM2* (which activates Ca^2+^ entry channels) and the downregulation of its negative regulator *DUSP4*. In contrast, in ABC-DLBCL, negative regulation of MAPK by CXCR4 signaling was indicated by the downregulation of *FYN* upstream of the MAPK signaling pathway, and the downregulation of calcium-dependent molecules such as *MFAP4.* In the GCB subtype, *CYLD* and *UBD* which activate NF-κB were downregulated in CXCR4^+^ compared to CXCR4^−^ DLBCL patients. In the ABC subtype, NF-κB pathway showed opposite regulations: NF-κB activators *CARD11* and *PIM2* were upregulated, whereas *TNFSF12*, *TNFSF8* and *IL12RB* were downregulated (Figure 4I, Supplemental Figures 3J-L). In CXCR4^+^ GCB-DLBCL, *PTPN6*/*PTN6* which modulates epidermal growth factor receptor was downregulated whereas in CXCR4^+^ ABC-DLBCL, *HILPDA* which activates vascular endothelial growth factor A was upregulated.

Pathway analysis (http://www.qiagen.com/ingenuity) indicated CXCR4 signatures were associated with functional networks of cell-to-cell signaling and interaction, immune cell trafficking, hematological system development and function, cellular growth and proliferation, cell death and survival (Supplemental Figure 5).

### Effect of CXCR4 inhibitor BKT140 on the growth of DLBCL cells

**Figure 5:**
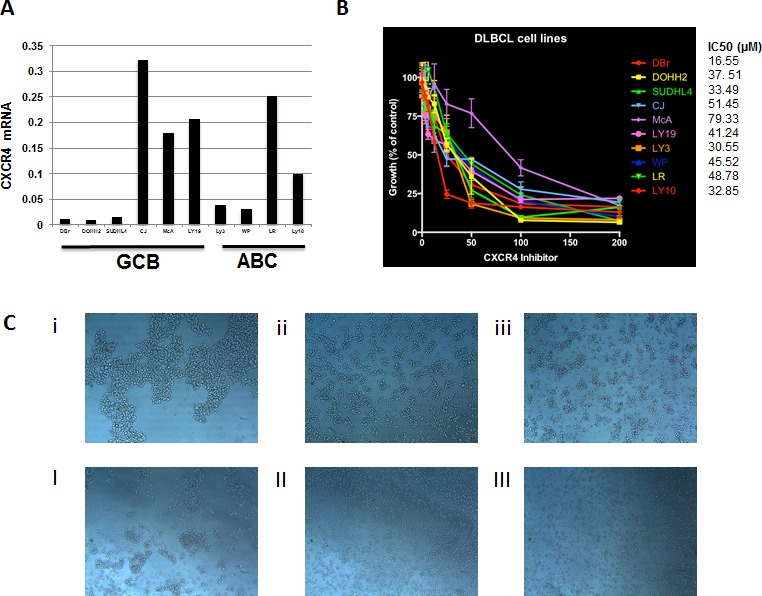
Effects of BTK140 on proliferation and the growth patterns of DLBCL cells (A) Expression levels of CXCR4 mRNA in DLBCL cell lines. (B) Ten DLBCL cell lines were treated with BTK140 in a dose-dependent manner. Cell proliferation was measured using ^3^H-thymiding incorporation assay after 72 hours incubation. Dates shown are the means and ranges of triplicate samples relative to control samples of three independent experiments. (C) The proliferation pattern of McA cells incubated without BKT140 (i), with 6.25uM BKT140 (ii), and with 50uM BKT140 (iii). (C) The proliferation pattern of LY19 incubated without BKT140 (I), with 6.25uM BKT140 (II), and with 50uM BKT140 (III).

We assessed the effect of the CXCR4 inhibitor BTK140 on growth of DLBCL cells. In 10 cell lines of either the GCB or ABC subtype, BKT140 treatment resulted in a significant dose-dependent growth inhibition in all 10 cell lines, with half maximal inhibitory concentration values ranging from 16.55 to 79.33 nM; however, the inhibition did not appear to depend on CXCR4 expression (Figure 5A-B). BTK140 indeed inhibit CXCR4-mediated cell adhesion, suggested by the alteration of growth patterns of DLBCL cells expressing high CXCR4 mRNA. The proliferation pattern of DLBCL cells changed from adhesive to discohesive after 48 hours of incubation with different concentrations of BKT140 (Figure 5C).

## DISCUSSION

The CXCR4/CXCL12 axis is essential for development, hematopoiesis, vascularization [4], and migration, homing and retention of stem cells. In the current study, CXCR4 expression was associated with poorer OS (*P*= .0016) and PFS (*P*= .0017) in a large cohort of 468 *de novo* DLBCL patients treated with R-CHOP (Figures 1H-I) and poorer therapy response in 236 GCB-DLBCLs. However, although univariate analysis of CXCR4 expression showed prognostic significance in both GCB and ABC subtypes, multivariate analysis indicated that CXCR4 expression was an independent prognostic factor for poorer PFS only in GCB-DLBCL patients. Furthermore, our data suggested that concurrent CXCR4 expression and *BCL2* translocation may represent another type of double-hit DLBCL with aggressive clinical courses. In ABC-DLBCL, Myc/Bcl-2 expression and TP53 mutations but not CXCR4 expression independently predicted poor survival. The lack of independent prognostic significance of CXCR4 expression in ABC-DLBCL was likely due to the tumor suppression function of WT-p53 whose expression was associated with CXCR4^+^ in ABC-DLBCL. It may also be attributed to the significantly (*P*= .0047) higher proportion of patients with an IPI>2 in ABC (68.4%) than in GCB (55%), as the prognostic significance and biologic impact of CXCR4 expression was only demonstrated in patients with low-risk IPI.

It is widely accepted that the CXCR4/CXCL12 axis underlies the decreased chemosensitivity and disease progression, by directing CXCR4-expressing tumor cells through concentration gradients of CXCL12 to reside in protective niche (such as BM and lymph nodes). Our results showed that CXCR4 expression had a significant prognostic impact in nodal DLBCL but not extranodal DLBCL. However, only 10.7% CXCR4^+^ patients showed BM involvement at diagnosis, and the adverse impact of CXCR4 expression in nodal DLBCL is independent of BM involvement, suggesting other malignant consequences besides BM homing ensuing CXCR4 expression in DLCBL. In patients without BM involvement, we surprisingly observed an inverse correlation between CXCR4 surface expression and *CXCL12* mRNA expression in stromal cells. This paradoxical phenomenon was also observed in primary kidney tumor tissues [43]. We speculate that the abnormal CXCR4^high^/CXCL12^low^ condition at the primary sites lead to the dissemination of CXCR4^+^ lymphoma cells to distant organs expressing higher CXCL12, which resulted in disease progression of CXCR4^+^ DLBCL. Supporting this hypothesis, CXCL12 expression was a strong independent prognostic biomarker for better survival in breast cancers [44], and administration of CXCL12 has been suggested as a potent inhibitor of colorectal and melanoma metastasis [45]. In addition, CXCR4^+^ demonstrated different prognostic values in different disease subsets although it consistently correlated with decreased *CXCL12* mRNA levels in these subsets (Figures 2, 3, Supplemental Figures 2, 3A-D). Therefore, *CXCL12* reduction alone may be insufficient to account for the CXCR4-associated disease progression.

Furthermore, our protein expression and GEP data suggested that the impact of CXCR4 on lymphoma relapse and progression of *de novo* DLBCL may be attributed to dysregulations in both the tumor microenvironment and the tumor cells themselves. These mechanisms used by CXCR4 for tumor cell survival may include reduced immune surveillance, increased tumor proliferation involving the upregulation of the Myc and PI3K/mTOR pathways, and blocked apoptosis involving Bcl-2 expression and the TP53 pathway. Previous studies showed that p53 negatively regulated expression of CXCR4 [2] (consistent with our results) and CXCL12 [46,47] (inconsistent with our mRNA results) abrogating the stromal cell-mediated chemoresistance. The role of CXCR4 signaling in promoting proliferation and survival was supported by the *in vitro* studies, where the high-affinity CXCR4 inhibitor BTK140 alone resulted in inhibited proliferation as well as inhibitory changes of adhesion and growth patterns in various DLBCL cell lines. These novel oncogenic mechanisms, in addition to the dissemination of CXCR4^+^ tumor cells to distant lymphatic tissues with high CXCL12 concentrations, may synergistically account for the CXCR4-mediated disease progression.

In cancer cells, CXCR4 expression can be caused by hypoxia, NF-κB activation, and ubiquitination inhibition [4]. In some patients of our cohort, increased CXCR4 expression may have resulted from reduced degradation, as suggested by decreased expression of *UBD/ubiquitin D* and the deubiquitinating enzyme *CYLD* in GCB-DLBCL and decreased E3 ubiquitin-protein ligase *MARCH2* in ABC-DLBCL. In ABC-DLBCL, upregulated *SUGT1*, which plays a role in ubiquitination and subsequent proteasomal degradation of target proteins, may counteract the CXCR4 increase. Hypoxia (as suggested by increased *HILPDA*) which is known for CXCR4 activation [3,4,9], may also be the causes of CXCR4 expression in ABC-DLBCL.

Cell-of-origin may as well explain the CXCR4^+^ phenotype. Some CXCR4^+^ GCB-DLBCLs may represent lymphoma cells arising from CXCR4^high^ centroblasts in the CXCL12-rich dark zone and CXCR4^−^ GCB-DLBCLs may be the transformed CXCR4^low^ centrocytes in the light zone, where B cells interact with follicular dendritic and T helper cells. This hypothesis is in line with the higher activation-induced cytidine deaminase levels, highly proliferative characteristics, and the lack of T cell signature in CXCR4^+^ patients [23,24], but is contradicted by the CXCR4^+^ associated low CXCL12 levels. A plausible explanation is that abnormal reduction in CXCL12 expression in lymph nodes (due to oncogenic mechanisms such as dysregulated TNF cytokines or Myc overexpression, or as the secondary event of *BCL2* translocation in the GC) initiated the tumorigenesis. This was selected for CXCR4^high^ lymphoma cells due to the dynamics of CXCL12/CXCR4 equilibrium and led to decreased T cell infiltration and deficient immune responses due to reduced chemoattraction, cooperating with the CXCR4-associated pro-survival signals. Decreased CXCL12 expression further led to dissemination of CXCR4^+^ tumor cells to distant lymphatic tissues with higher CXCL12 expression [28, 51]. Therefore, the abnormal CXCL12/CXCR4 levels may be relevant for both lymphomagenesis and disease progression.

In conclusion, our results indicated CXCR4 expression was associated with poorer clinical outcomes in DLBCL and independently predicted disease progression in GCB-DLBCL. The underlying mechanisms whereby CXCR4 exerts its prognostic impact may include tumor growth promotion, apoptosis inhibition, decreased T cell infiltration and immune responses, and tumor cell dissemination to distant organs/tissues. These results could help stratify DLBCL and gain insight of molecular events that function as therapeutic targets.

## PATIENTS AND METHODS

### Patients

The training study consisted of 468 patients with *de novo* DLBCL diagnosed between 2000 and 2010 and treated with R-CHOP (median age: 63 years). The diagnostic criteria, review process, eligibility and exclusion criteria, cell-of-origin classification as either GCB or ABC subtype via GEP or IHC algorithms have been described previously [38,41,48]. At last follow-up, 176 of 468 (37.6%) patients had died. The median follow-up for the 292 censored patients was 48.7 months. For validation, an independent cohort of 275 *de novo* DLBCLs diagnosed between 2002 and 2007 and treated with R-CHOP was used, with median follow-up of 50 months. This study was conducted in accordance with the Declaration of Helsinki and was approved as being of minimal to no risk or as exempt by the Institutional Review Boards of all participating centers, including The University of Texas MD Anderson Cancer Center.

### CXCR4 cell surface expression and other pathological experiments

IHC analyses for CXCR4 expression using polyclonal anti-CXCR4 antibody (Abcam) and antihuman CXCR4 mAb (R&D Systems) were performed on tissue microarrays of formalin-fixed, paraffin-embedded (FFPE) lymphoma samples and was assessed by three pathologists blinded from clinical outcomes with similar results. The inter-observer agreement was 98%, and the disagreement was resolved by joint review at a multi-headed microscope.

IHC of other biomarkers using respective monoclonal antibodies, fluorescence *in situ* hybridization to detect *MYC*, *BCL6*, and *BCL2* translocations, and *TP53* resequencing using p53 AmpliChip have been described previously [38-41,48,49].

### Gene expression profiling

GEP by the Affymetrix GeneChip Human Genome U133 Plus 2.0 array was performed using total RNAs extracted from FFPE tissues as previously described [38-41,48,49]. Normalized microarray data were analyzed for differential gene expression between the CXCR4^+^ and CXCR4^−^ groups. Univariate analysis using a *t* test was performed to identify differentially expressed genes. The *P* values obtained via multiple *t*-tests were corrected for FDRs using the beta-uniform mixture method. Differentially expressed genes were identified at various FDRs with different *P* value cutoffs. Pathway analysis for the identified DEGs was performed using Ingenuity® Pathway Analysis (IPA^®^, http://www.qiagen.com/ingenuity) software program.

### Effect of CXCR4 inhibitor BTK140 *in vitro*

The inhibitory effect of BTK140 (kind gift from BioLineRx Ltd, Jerusalem, Israel) was evaluated in 10 DLBCL cell lines that were either the GCB (DBr, DOHH2, SUDHL4, CJ, McA, LY19) or ABC (LY3, WP, LR, and LY10) subtype that were cultured and maintained in RPMI 1640 (Life Technologies, Rockville, MD, USA) and 15% fetal calf serum (HyClone, Logan, UT, USA). [^3^H] thymidine proliferation assays *in vitro* were performed as described previously [50]. Different concentrations of BKT140 were used: 3.125 μM, 6.25 μM, 12.5 μM, 25 μM, 50 μM, 100 μM, and 200 μM. Cell proliferation was measured using ^3^H-thymidine incorporation assays after 72 hours of incubation.

### Statistical analysis

The clinicopathologic features of CXCR4^+^ and CXCR4^−^ DLBCL patients at the time of presentation were compared using the chi-square test. Overall survival was calculated from the time of diagnosis to death from any cause or last follow-up. Progression-free survival was calculated from the time of diagnosis to disease progression, relapse, or death from any cause.^21^ Patients who were alive and/or had no disease progression were censored at last follow-up. Survival analysis was performed using the Kaplan–Meier method with GraphPad Prism 6 (GraphPad Software, San Diego, CA), and differences were compared using the log-rank (Mantel-Cox) test. Multivariate survival analysis was performed using the Cox proportional hazards regression model with the SPSS statistics software program (version 19.0; IBM Corporation, Armonk, NY). All differences with *P* ≤ .05 were considered statistically significant.

## SUPPLEMENTARY MATERIAL FIGURES


