# Supplementary material for: Dysregulated CXCR4 expression promotes lymphoma cell survival and independently predicts disease progression in germinal center B-cell-like diffuse large B-cell lymphoma
Source: Oncotarget. 2015 Jan 21;6(8):5597–614. doi: 10.18632/oncotarget.3343 (PMC4467389; doi:10.18632/oncotarget.3343)
Supplement: Supplementary file 1 [file oncotarget-06-5597-s001.pdf]

Dysregulated CXCR4 expression promotes lymphoma cell survival and independently predicts disease progression in germinal center B-cell-like diffuse large B-cell lymphoma

Supplementary Material

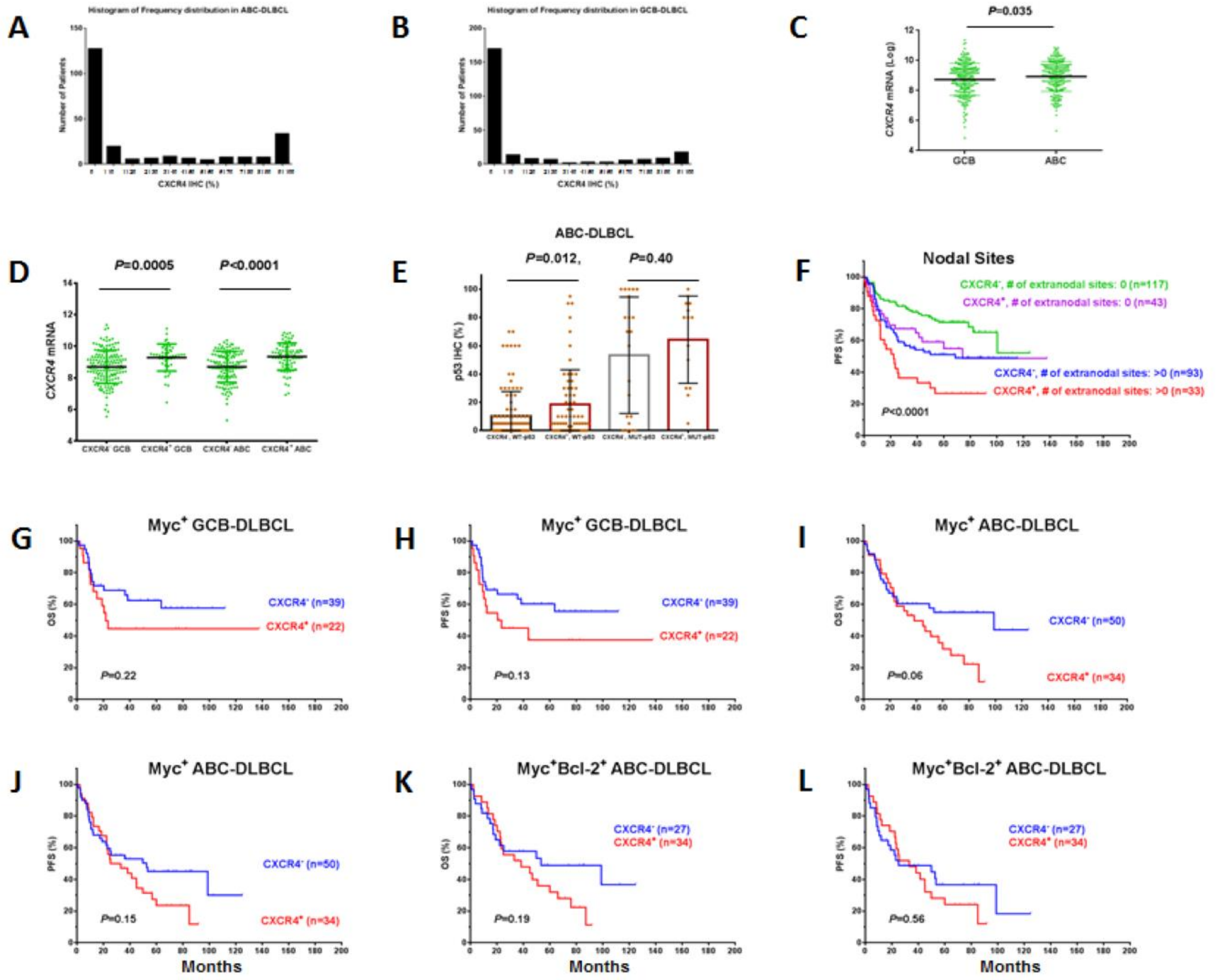

Supplemental Figure 1: (A-B) Histograms showing the distribution of CXCR4 expression frequency in the ABC- and GCB-DLBCL subcohort. X-axis, percentage of immunopositive cells in tumors; Y-axis, numbers of DLBCL patients. **(C)** ABC-DLBCL compared to GCB-DLBCL had increased CXCR4 mRNA expression. **(D)** Correlation between CXCR4 surface expression and mRNA levels. **(E)** CXCR4 expression was associated with increased WT-p53 expression in ABC-DLBCL. **(F)** Impact of CXCR4 expression on survival of nodal LDBCL with or without extranodal involvement. **(G-J)** Impact of CXCR4 expression on Myc-overexpressing GCB- and ABC-DLBCL. **(K-L)** Impact of CXCR4 expression in ABC-DLBCL with concurrent Myc/Bcl-2 expression.

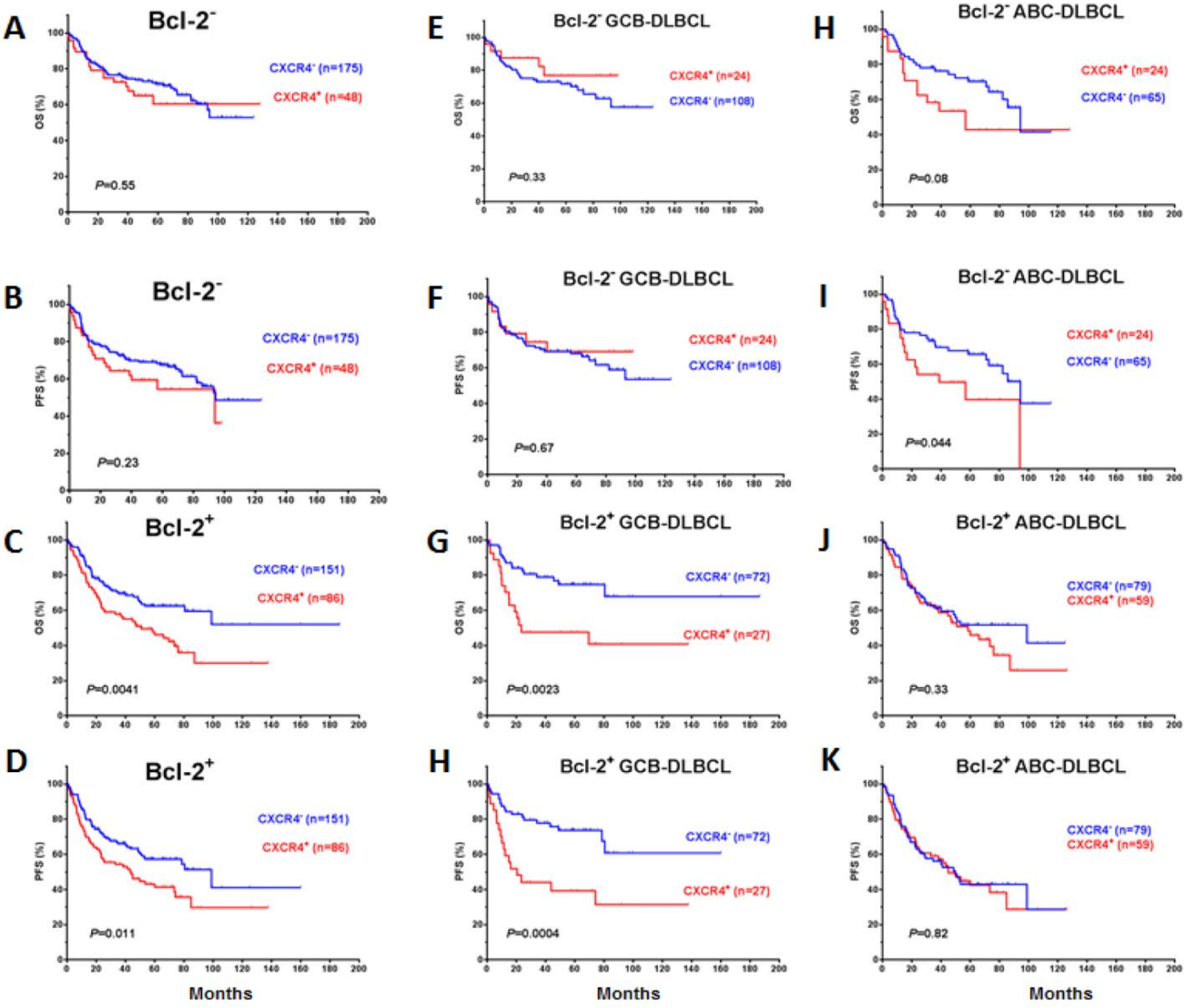

Supplemental Figure 2: Prognostic significance of CXCR4 expression in patients with or without Bcl-2 expression.

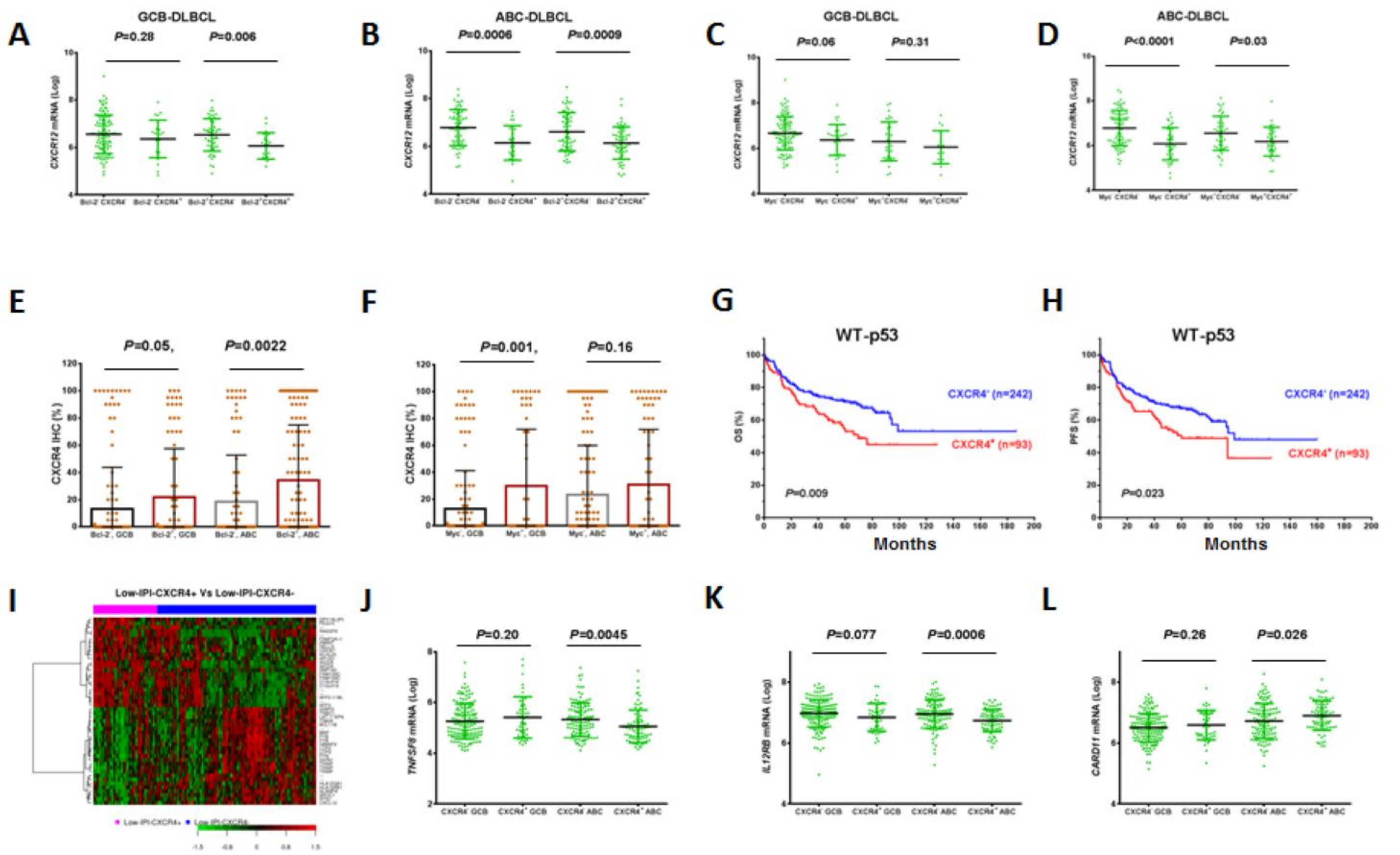

Supplemental Figure 3: (A-D) CXCR4 cell surface expression correlated with decreased *CXCL12* mRNA levels, independent of Bcl-2 and Myc overexpression status. **(E-F)** Myc/Bcl-2 expression was associated with CXCR4 cell surface expression, both in GCB- and ABC-DLBCL (*P* value for Myc^{+} ABC-DLBCL was not significant). (**G-H**) CXCR4 expression correlated with significantly poor OS and PFS in DLBCL patients with WT-p53. (**I**) Heatmaps and differentially expressed genes between CXCR4^{+} and CXCR4^{-} DLBCL patients with an IPI of 2 or lower. (**J-L**) Differentially regulation of *TNFSF8*, *IL12RB*, and *CARD11* by CXCR4 signaling between GCB- and ABC-DLBCL.

Validation set

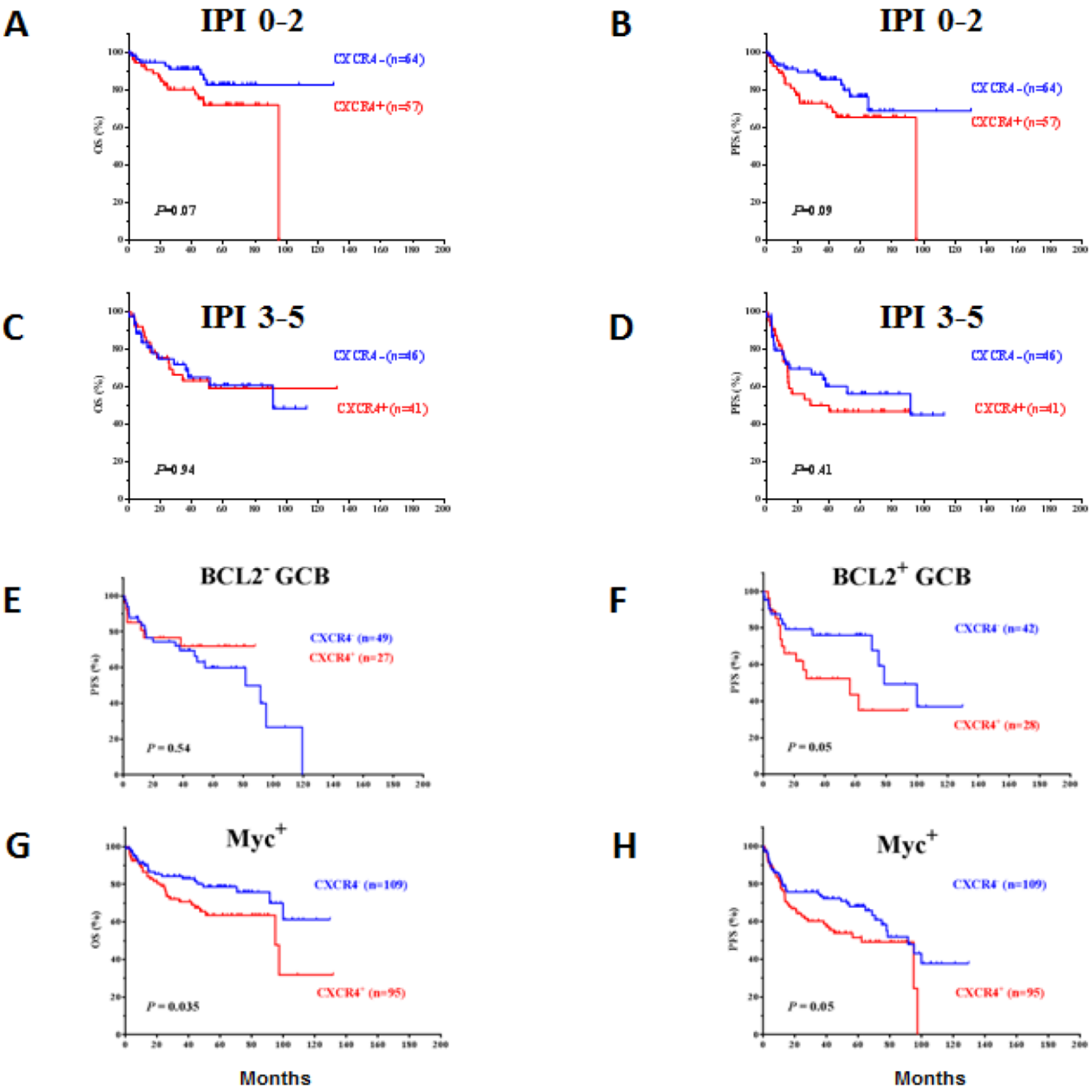

Supplemental Figure 4: Prognostic impact of CXCR4 expression in the validation set of 275 *de novo* DLBCL patients.

In DLBCL CXCR4⁺ associated Network 1: Cellular Movement, Hematological System Development and Function, Immune Cell Trafficking

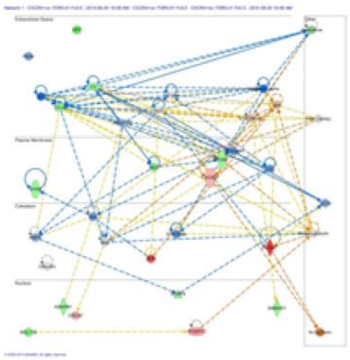

In DLBCL CXCR4⁺ associated Network 2: Cellular Movement, Hematopoiesis, Immune Cell Trafficking

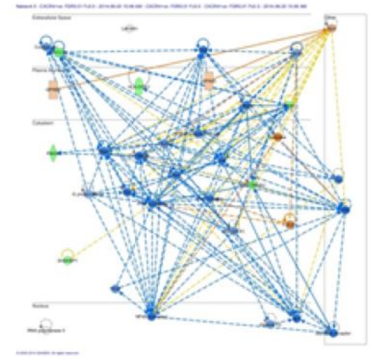

In DLBCL CXCR4⁺ associated Network 3: Hematological System Development and Function, Tissue Morphology, Hematopoiesis

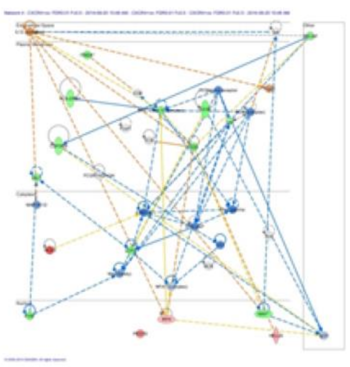

In GCB-DLBCL CXCR4⁺ associated Network 1: Cardiovascular System Development and Function, Cellular Function and Maintenance, Cellular Growth and Proliferation

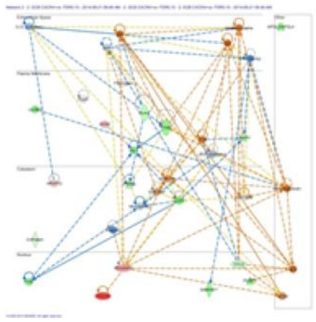

In GCB-DLBCL CXCR4⁺ associated Network 2: Cell-To-Cell Signaling and Interaction, Nervous System Development and Function, Gastrointestinal Disease

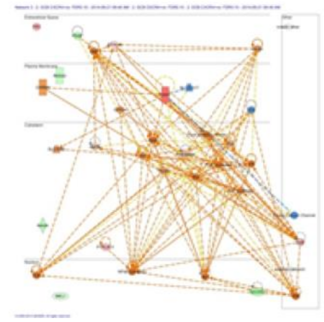

In ABC-DLBCL CXCR4⁺ associated Network 1: Cell-To-Cell Signaling and Interaction, Hematological System Development and Function, Cell Death and Survival

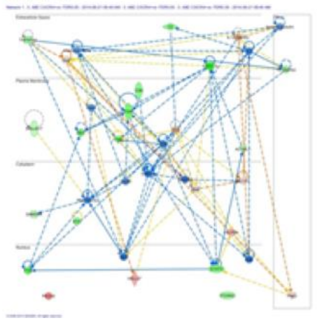

In ABC-DLBCL CXCR4⁺ associated Network 2: Hematological System Development and Function, Humoral Immune Response, Cancer

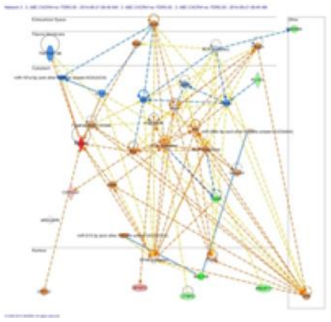

Supplemental Figure 5: Associated networks for CXCR4 signatures by IPA software analysis.
